# Chronic ACL-injured patients show increased medial and global anterior tibial subluxation measured on 1-year postoperative MR images after primary single-bundle ACL reconstruction

**DOI:** 10.1186/s13018-023-04028-5

**Published:** 2023-08-03

**Authors:** Zhi-yu Zhang, Wei-li Shi, Wen-bin Bai, Ling-yu Meng, Qing-yang Meng, Jian-quan Wang, Cheng Wang

**Affiliations:** 1 Department of Sports Medicine, Peking University Third Hospital, Institute of Sports Medicine of Peking University, No. 49, Huayuanbei Road, Haidian District, Beijing, China; 2 Beijing Key Laboratory of Sports Injuries, Beijing, China; 3 Engineering Research Center of Sports Trauma Treatment Technology and Devices, Ministry of Education, Beijing, China; 4 School of Basic Medical Science, Peking University, Beijing, China

**Keywords:** Anterior cruciate ligament, Anterior tibial subluxation, Internal rotational tibial subluxation, Tibiofemoral position, Chronicity, MRI

## Abstract

**Background:**

The association between chronic anterior cruciate ligament (ACL) injury and inferior postoperative outcomes following ACL reconstruction (ACLR) has been highlighted in the literature. However, the inclusion of postoperative radiological assessments in previous studies has been limited. The aim of this study is to investigate whether chronic ACL injury is associated with an inferior tibiofemoral position measured on magnetic resonance (MR) images after primary ACLR.

**Methods:**

A total of 62 patients that underwent primary ACLR were included in this study based on the time from injury to surgery, namely the acute ACL-injured group (within 6 weeks) and the chronic ACL-injured group (more than 1 year) and were matched 1:1 according to sex, age (± 2 years), and time from surgery to follow-up (± 3 months). Patient demographics, surgical records and follow-up data were retrieved and analyzed. The altered tibiofemoral position was measured quantitatively on preoperative and at least 1-year postoperative MR images and compared between the two groups, including the lateral, medial and global anterior tibial subluxation (LATS, MATS and GATS) and internal rotational tibial subluxation (IRTS).

**Results:**

No significant differences in preoperative LATS, MATS, GATS or IRTS were identified between the acute and chronic ACL-injured groups. The chronic ACL-injured patients showed significantly increased postoperative MATS (*p* = 0.001) and GATS (*p* = 0.012), while no significant difference was identified in postoperative LATS or IRTS. Multivariate linear regression analyses showed that chronic ACL injury resulted in an estimated increase of 2.0 mm in postoperative MATS (*p* = 0.012) and 1.9 mm in postoperative GATS (*p* = 0.040). A significant improvement in postoperative LATS was observed in the acute ACL-injured group (*p* = 0.044) compared to preoperative LATS, while no improvements in these MRI measurements were observed in the chronic ACL-injured group.

**Conclusion:**

Chronic ACL-injured patients showed increased MATS and GATS measured on 1-year postoperative MR images after primary single-bundle ACL reconstruction, while no difference was identified in rotational tibiofemoral position. The acute ACL-injured group demonstrated a significant improvement in postoperative LATS, whereas no improvements were observed in the chronic ACL-injured group.

*Level of evidence* Level III.

**Supplementary Information:**

The online version contains supplementary material available at 10.1186/s13018-023-04028-5.

## Introduction

Chronic anterior cruciate ligament (ACL) injury can result in meniscal and chondral lesions, which can lead to the progression of knee osteoarthritis [1–5]. Patients with chronic ACL injury who undergo isolated ACL reconstruction (ACLR) may have lower postoperative patient-reported outcomes (PROs) compared to those with acute ACL injury [6] and may also have a higher early graft failure rate at 6 months [7]. To address these issues, some researchers have suggested performing ACLR with anterolateral complex (ALC) augmentation for patients with chronic ACL injury to restore rotational instability and improve postoperative outcomes [8]. However, the surgical indications for ALC augmentation in chronic ACL injury remain a topic of debate, and there is still a lack of "reliable" methods to confirm whether a patient with chronic ACL injury should be diagnosed with rotational instability. Therefore, investigating the preoperative and postoperative characteristics of patients with chronic ACL injury holds clinical relevance, as it can contribute to preoperative planning and decision-making. Although many studies have evaluated the PROs and graft failure rates of patients undergoing ACLR [6, 7], few have included postoperative radiological evaluations, possibly due to limited patient compliance or financial constraints. Thus, there is a significant gap in our understanding of the radiological outcomes of postoperative patients.

Radiological characteristics of ACL injury can be quantitatively described in terms of the altered anteroposterior and rotational tibiofemoral position [9–11]. Previous studies have reported that patients with ACL injury showed increased anterior tibial subluxation (ATS) [10, 12–14] and internal rotational tibial subluxation (IRTS) [10], as measured on magnetic resonance (MR) images, when compared to those with an intact ACL. However, it remains unknown whether these subluxations can be restored after ACLR, particularly in relation to the effect of chronicity on postoperative variation of the tibiofemoral position.

In this study, we quantitatively measured the altered tibiofemoral position on preoperative and 1-year postoperative MR images in patients that underwent primary ACLR. The aim of this study was to determine whether chronic ACL injury was associated with an inferior postoperative tibiofemoral position. Our hypothesis was that patients with chronic ACL injury would show an increased postoperative ATS and IRTS compared to those with acute ACL injury.

## Materials and methods

### Patients

This study was approved by the ethics committee of our institute. A total of 151 patients with available preoperative and at least 1-year postoperative MR imaging (MRI) scans at our institute were retrospectively reviewed from among 765 patients that underwent anatomic single-bundle ACL reconstruction from 2012 to 2018 with autologous hamstring tendon grafts. Patient demographics, surgical records and postoperative data were retrieved and analyzed.

Patients were divided into two groups based on the time from injury to surgery, namely the acute ACL-injured group and the chronic ACL-injured group, and were matched 1:1 according to sex, age (± 2 years), and time from surgery to follow-up (± 3 months). The chronic ACL-injured group included 31 patients meeting the following criteria: (1) time from injury to surgery more than 1 year; (2) primary ACLR without concomitant injuries to the posterior cruciate ligament (PCL) or collateral ligaments; (3) no history of patellar dislocations; (4) no history of other knee surgery; and (5) no graft failure. The acute ACL-injured group included 31 patients meeting the same criteria, except that the time from injury to surgery was within 6 weeks. For patients with concomitant meniscal injuries, the treatment approach was determined based on the findings during arthroscopy. In such cases, either suture repair or meniscoplasty procedures were routinely performed. All patients included in this study underwent preoperative MR scans at our institute within 2 weeks before their surgeries, ensuring that the imaging data used for analysis were obtained close to the surgical procedures. Moreover, all MR scans included in this study were obtained with patients positioned in a supine posture, with a neutral rotational position of the lower limb. The scans were performed by experienced radiographic technicians following our institutional standard protocol.

### MRI measurements

MRI scans were performed using a GE Discovery MR750 3.0-T System (PrizMed Imaging, Unit A Willowick, Ohio, USA) with a slice thickness of 3 mm for all sequences. Patients were positioned supine with their knees fully extended in the neutral position. The measuring protocol for ATS in the lateral compartment (LATS) and medial compartment (MATS) utilized the longitudinal tibial axis as the reference line, and has been previously reported to have good-to-excellent reliability [9–11, 15]. The longitudinal tibial axis was determined on the central slice that showed the intercondylar eminence [15]. In the lateral compartment, LATS was measured on the central lateral slice showing the most medial section of the fibula at the tibiofibular joint. A line parallel to the longitudinal tibial axis was drawn at the posterior margin of the posterior femoral condyle and the tibial plateau. The distance between these two lines was measured as the LATS. Similarly, the same protocol was performed on the central medial slice that showed the insertion of the medial gastrocnemius tendon on the femur for the measurement of MATS. The global anterior tibial subluxation (GATS), which represents the overall anteroposterior tibiofemoral position, was calculated by averaging the values of LATS and MATS [14]. Internal rotational tibial subluxation (IRTS) was determined by subtracting MATS from LATS, indicating the internal rotational tibiofemoral position [10]. To ensure the reliability of the measurements in this study, 10 knees from each group were randomly selected for intra- and interobserver reliability testing, showing good-to-excellent reliability.

### Statistical analysis

Statistical analyses were performed using R for Mac OS X GUI version 4.0.0 (R Foundation for Statistical Computing, Vienna, Austria). The normality of data distributions and equality of variances were checked using the Shapiro–Wilk test and the Bartlett test, respectively. Unpaired t-tests, Mann–Whitney tests (i.e., Wilcoxon rank-sum tests), and Fisher's exact tests were performed to compare variables between the acute and chronic ACL-injured groups. Multivariate linear regression models were established to control for confounding variables and determine the association between the chronicity of ACL injury and postoperative MRI measurements. The paired t-test was used for longitudinal comparisons between preoperative and postoperative MRI measurements respectively in the two groups. A significance level of *P* < 0.05 was considered statistically significant. Power analysis and sample size calculation were performed using R for Mac (version 4.0.0). A two-sample, two-sided model with a Type I error of 5% (α = 0.05) was employed. The mean and standard deviation values of MATS in the two groups were used to estimate the effect size. The estimated Cohen's d value was 0.743, and the calculated power was 0.821 for a total of 62 patients included in this study. With a desired power of 0.8 (β = 0.20), a minimum of 29.4 patients should be included in each group.

## Results

**Fig. 1:**
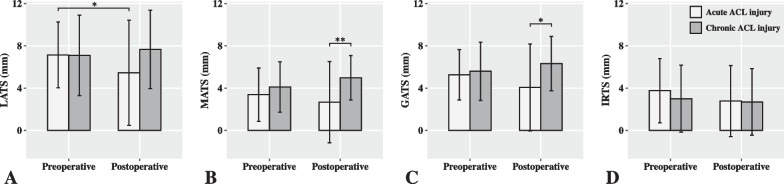
Comparisons of LATS (**A**), MATS (**B**), GATS (**C**) and IRTS (**D**) between acute and chronic ACL-injured group. ACL, anterior cruciate ligament; GATS, global anterior tibial subluxation; IRTS internal rotational tibial subluxation; LATS, lateral anterior tibial subluxation; MATS, medial anterior tibial subluxation

There were no significant demographic differences between the acute and chronic ACL-injured groups, including sex, age, body mass index (BMI), Beighton score, affected side, and time from surgery to follow-up (Table 1). Intraoperative arthroscopic findings showed a higher frequency of medial meniscal injury (*p* = 0.023) in the chronic ACL-injured group compared to the acute ACL-injured group (Table 1). Preoperative MRI measurements showed no significant differences in LATS, MATS, GATS or IRTS between the acute and chronic ACL-injured groups (Table 2, Fig. 1).

**Table 1:** Patient demographics and arthroscopic findings

	Acute ACL-injured (n = 31)	Chronic ACL-injured (n = 31)	*p* value
Sex, male:female	20:11	20:11	1
Age, mean ± SD (range), year	28.3 ± 7.4 (16, 47)	28.7 ± 7.4 (18, 47)	0.849
BMI, mean ± SD (range)	24.1 ± 3.5 (18.3, 33.6)	24.6 ± 3.2 (18.0, 31.2)	0.518
Beighton Score, median [IQR]	1 [0, 3.5]	2 [0, 5]	0.220
Affected side, left:right	12:19	7:24	0.168
Time from injury to surgery, median [IQR], day	30 [20.5, 40]	1080 [480, 1800]	** < 0.001**
Time from injury to preoperative MRI, median [IQR], day	17 [2, 28]	1077 [467.5, 1784.5]	** < 0.001**
Time from surgery to follow-up, median [IQR], month	13 [12, 13.5]	13 [12, 14.5]	0.855
Articular cartilage lesion, frequency (percentage)	20 (64.5)	27 (87.1)	0.075
Lateral meniscal injury, frequency (percentage)	11 (35.5)	16 (51.6)	0.200
Anterior horn	1 (3.2)	4 (12.9)	0.354
Body	4 (12.9)	7 (22.6)	0.506
Posterior horn	7 (22.6)	12 (38.7)	0.168
Lateral meniscal surgery, frequency (percentage)			
Suture repair	6 (19.4)	4 (12.9)	0.730
Meniscoplasty	5 (16.1)	12 (38.7)	**0.046**
Medial meniscal injury, frequency (percentage)	18 (58.1)	27 (87.1)	**0.023**
Anterior horn	1 (3.2)	0 (0.0)	1
Body	4 (12.9)	11 (35.5)	0.075
Posterior horn	18 (58.1)	25 (80.6)	0.054
Medial meniscal surgery, frequency (percentage)			
Suture repair	12 (38.7)	14 (45.2)	0.607
Meniscoplasty	6 (19.4)	13 (41.9)	0.054

Bold values indicate *p* value < 0.05

*ACL* anterior cruciate ligament, *BMI* body mass index, *IQR* interquartile range, *MRI* magnetic resonance imaging, *SD* standard deviation

**Table 2:** MRI measurements in the acute and chronic ACL-injured group

mean ± SD (range), mm	Acute ACL-injured (n = 31)	Chronic ACL-injured (n = 31)	*p* value
Preoperative measurements			
LATS	7.1 ± 3.1 (1.4, 13.6)	7.1 ± 3.8 (−0.5, 14.3)	0.959
MATS	3.4 ± 2.5 (−0.6, 8.3)	4.1 ± 2.4 (0.0, 8.4)	0.253
GATS	5.3 ± 2.4 (1.1, 10.2)	5.6 ± 2.8 (−0.3, 11.2)	0.609
IRTS	3.8 ± 3.0 (−3.3, 9.1)	3.0 ± 3.2 (−2.9, 9.6)	0.336
Postoperative measurements			
LATS	5.5 ± 5.0 (−6.8, 17.5)	7.7 ± 3.7 (0.3, 13.8)	0.051
MATS	2.7 ± 3.8 (−2.4, 13.3)	5.0 ± 2.1 (0.7, 9.8)	**0.001**
GATS	4.1 ± 4.1 (−4.3, 15.2)	6.3 ± 2.6 (0.5, 10.4)	**0.012**
IRTS	2.8 ± 3.4 (−4.9, 8.2)	2.7 ± 3.2 (−4.6, 9.6)	0.922

Bold values indicate *p* value < 0.05

*ACL* anterior cruciate ligament, *GATS* global anterior tibial subluxation, *IRTS* internal rotational tibial subluxation, *LATS* lateral anterior tibial subluxation, *MATS* medial anterior tibial subluxation, *MRI* magnetic resonance imaging, *SD* standard deviation

Postoperative MRI measurements showed that the chronic ACL-injured group had significantly increased MATS (*p* = 0.001) and GATS (*p* = 0.012) compared to the acute ACL-injured group, but no significant difference was observed in postoperative LATS or IRTS (Table 2, Fig. 1). These results suggest that chronic ACL injury is associated with worse postoperative anteroposterior tibiofemoral position but not with rotational tibiofemoral position. Multivariate linear regression models were used to further confirm these findings, indicating that chronic ACL injury was associated with postoperative MATS and GATS, resulting in an estimated increase of 2.0 mm in MATS (*p* = 0.012) and 1.9 mm in GATS (*p* = 0.040), but not with postoperative LATS or IRTS (Table 3). Furthermore, male sex was associated with a decrease in postoperative MATS (*p* = 0.001) and an increase in IRTS (*p* = 0.029) (Table 3). The complete models can be found in the Additional file 1: Tables S1–S4.

**Table 3:** Multivariate linear regression analyses for postoperative MRI measurements^a^

Variables (n = 62)	Estimate (mm)	SE	95% CI	*P* value
Dependent variable: LATS				
(Intercept)^b^	10.339	4.545	(1.222, 19.456)	**0.027**
Dependent variable: MATS				
Male sex	−3.042	0.886	(−4.819, −1.264)	**0.001**
Chronic ACL injury	2.024	0.775	(0.470, 3.578)	**0.012**
Dependent variable: GATS				
(Intercept)^b^	7.975	3.434	(1.087, 14.863)	**0.024**
Chronic ACL injury	1.941	0.921	(0.093, 3.789)	**0.040**
Dependent variable: IRTS				
Male sex	2.260	1.010	(0.234, 4.285)	**0.029**

Bold values indicate *p* value < 0.05

*ACL* anterior cruciate ligament, *CI* confidence interval, *GATS* global anterior tibial subluxation, *IRTS* internal rotational tibial subluxation, *LATS* lateral anterior tibial subluxation, *MATS* medial anterior tibial subluxation, *MRI* magnetic resonance imaging, *Std* standard

^a^The variables that did not show statistical significance in the model are not included in the presentation. The complete models can be found in the Additional file 1: Tables S1–S4)

^b^Intercept refers to the constant term of the linear regression model

To evaluate the postoperative improvement of altered tibiofemoral position, longitudinal comparisons were performed between preoperative and postoperative MRI measurements. The results showed a significant improvement in postoperative LATS in the acute ACL-injured group (*p* = 0.044) compared to preoperative LATS (Table 4, Fig. 1A). Although no statistically significant improvement was identified in postoperative MATS (*p* = 0.310), GATS (*p* = 0.094), or IRTS (*p* = 0.084) in the acute ACL-injured group, their mean values were decreased compared to the preoperative measurements, suggesting a trend of improvement (Table 4, Fig. 1). However, in the chronic ACL-injured group, the mean values of postoperative LATS, MATS, and GATS were increased compared to the preoperative measurements, indicating a lack of improvement (Table 4, Fig. 1).

**Table 4:** Longitudinal comparisons of MRI measurements

mean ± SD (range), mm	Preoperative	Postoperative	*p* value
Acute ACL-injured group			
LATS	7.1 ± 3.1 (1.4, 13.6)	5.5 ± 5.0 (−6.8, 17.5)	**0.044**
MATS	3.4 ± 2.5 (−0.6, 8.3)	2.7 ± 3.8 (−2.4, 13.3)	0.310
GATS	5.3 ± 2.4 (1.1, 10.2)	4.1 ± 4.1 (−4.3, 15.2)	0.094
IRTS	3.8 ± 3.0 (−3.3, 9.1)	2.8 ± 3.4 (−4.9, 8.2)	0.084
Chronic ACL-injured group			
LATS	7.1 ± 3.8 (−0.5, 14.3)	7.7 ± 3.7 (0.3, 13.8)	0.473
MATS	4.1 ± 2.4 (0.0, 8.4)	5.0 ± 2.1 (0.7, 9.8)	0.055
GATS	5.6 ± 2.8 (−0.3, 11.2)	6.3 ± 2.6 (0.5, 10.4)	0.176
IRTS	3.0 ± 3.2 (−2.9, 9.6)	2.7 ± 3.2 (−4.6, 9.6)	0.687

Bold values indicate *p* value < 0.05

*ACL* anterior cruciate ligament, *GATS* global anterior tibial subluxation, *IRTS* internal rotational tibial subluxation, *LATS* lateral anterior tibial subluxation, *MATS* medial anterior tibial subluxation, *MRI* magnetic resonance imaging, *SD* standard deviation

## Discussion

The most important finding of the present study was that patients with chronic ACL injury (time from injury to surgery more than 1 year) showed an increased 1-year postoperative MATS and GATS but a similar IRTS compared to the acute ACL injury (time from injury to surgery less than 6 weeks), indicating that the chronicity of ACL injury was associated with an inferior postoperative anteroposterior tibiofemoral position, but not with the postoperative rotational tibiofemoral position. Moreover, the acute ACL-injured group demonstrated a significant improvement in postoperative LATS, whereas no improvements in these MRI measurements were identified in the chronic ACL-injured group, highlighting the importance of surgical intervention after acute ACL injury.

There has been a growing interest in studies on chronic ACL injury due to reports of lower postoperative patient-reported outcomes (PROs) and a higher early graft failure rate. Previous studies have reported that chronic ACL injury is associated with a higher incidence of high-grade pivot shift [16] and that a combined lateral extra-articular tenodesis (LET) with ACL reconstruction may restore rotational stability in a chronic ACL-injured cadaveric model [17]. However, the evidence is insufficient to make a clinical decision on whether to perform a combined ALC augmentation for patients with chronic ACL injury. Furthermore, the technical complexity of the surgery may result in complications without improved outcomes [8]. This is probably because preoperative patients with chronic ACL injury have shown a more symmetrical gait, improved muscle strength [18], and better functional scores compared with acute patients [18, 19]. In this study, we found that chronic ACL-injured patients showed an increased anterior tibial subluxation, especially in the medial compartment, indicated by the higher MATS and GATS but a similar IRTS at least 1 year after primary single-bundle ACLR compared to acute ACL-injured patients. Therefore, the inferior postoperative outcomes reported in previous studies could be attributed to the increased postoperative anterior tibial subluxation observed in chronic ACL-injured patients, whereas no evidence was found to suggest that these patients would have an inferior rotational tibiofemoral position postoperatively. Additional indications may still be required to perform a combined ALC augmentation for chronic ACL-injured patients. Future research should focus on the association between PROs or the failure rate and the postoperative altered tibiofemoral position to provide more direct evidence.

The longitudinal comparisons of MRI measurements showed a significant improvement in postoperative LATS and a trend of improvement in postoperative MATS, GATS, and IRTS in the acute ACL-injured group. In contrast, no improvement or trend of improvement was observed in the chronic ACL-injured group (Table 4). These findings suggest that patients with chronic ACL injury may experience inferior improvement of the altered tibiofemoral positions after ACLR, highlighting the importance of surgical intervention after acute ACL injury.

Additionally, our analysis revealed that male patients were associated with a decrease in postoperative MATS and an increase in IRTS, whereas no significant associations were found in the postoperative LATS or GATS. This sex difference may be attributed to the fact that female patients generally have less muscle strength, which may lead to an increased tendency for the MATS to rise due to the lack of muscular protection.

### Limitations

This study has several limitations. Firstly, the sample size was limited due to the requirement for at least 1-year postoperative MRI scans, which led to restrictions in patient inclusion due to compliance issues and financial constraints. Secondly, the 1-year postoperative data only represents short-term postoperative outcomes, highlighting the need for future studies with long-term follow-up. Additionally, this retrospective study only focused on postoperative MRI measurements, and did not include other clinical outcomes such as patient-reported outcomes, graft failure rates, muscle strength improvement, or physical examinations. Lastly, the accuracy of the MRI measurements may have been influenced by various factors, such as the 3-mm slice thickness, the height of the tibia available on the images, and the knee flexion angle at the time of the MRI scan, which could potentially introduce bias into the results. Nevertheless, all MR scans used in this study were obtained using a standardized imaging protocol at our institute, which helps to reduce these potential random errors.

## Conclusion

Chronic ACL-injured patients showed increased MATS and GATS measured on 1-year postoperative MR images after primary single-bundle ACL reconstruction, while no difference was identified in rotational tibiofemoral position. The acute ACL-injured group demonstrated a significant improvement in postoperative LATS, whereas no improvements were observed in the chronic ACL-injured group.

### Supplementary Information

**Additional file 1**. The supplementary tables and figure provide detailed data on the results of the multivariate linear regression analyses.

## Data Availability

All data generated or analyzed during this study are included in this published article.

## Abbreviations

ACL: Anterior cruciate ligament
ACLR: Anterior cruciate ligament reconstruction
ATS: Anterior tibial subluxation
GATS: Global anterior tibial subluxation
IRTS: Internal rotational tibial subluxation
LATS: Lateral anterior tibial subluxation
MATS: Medial anterior tibial subluxation
MRI: Magnetic resonance imaging

## References

[1] Tanaka Y, Kita K, Takao R, et al. Chronicity of anterior cruciate ligament deficiency, part 1: effects on the tibiofemoral relationship before and immediately after anatomic ACL reconstruction with autologous hamstring grafts. Orthop J Sports Med. 2018;6. 10.1177/2325967117750813 10.1177/2325967117750813 PMC5784495 29383322

[2] Lohmander LS, Englund PM, Dahl LL (2007). The long-term consequence of anterior cruciate ligament and meniscus injuries: osteoarthritis. Am J Sports Med.

[3] Friel NA, Chu CR (2013). The role of ACL injury in the development of posttraumatic knee osteoarthritis. Clin Sports Med.

[4] Alonso B, Bravo B, Mediavilla L (2020). Osteoarthritis-related biomarkers profile in chronic anterior cruciate ligament injured knee. Knee.

[5] Rajput V, Haddad FS (2022). Is the die cast? Anterior cruciate ligament injury and osteoarthritis. Bone Joint J.

[6] Goradia VK, Grana WAA (2001). Comparison of outcomes at 2 to 6 years after acute and chronic anterior cruciate ligament reconstructions using hamstring tendon grafts. Arthroscopy.

[7] Tanaka Y, Kita K, Takao R, et al. Chronicity of anterior cruciate ligament deficiency, part 2 radiographic predictors of early graft failure*.* Orthop J Sports Med. 2018;6. 10.1177/2325967117751915. 10.1177/2325967117751915 PMC5818097 29479543

[8] Zaffagnini S, di Sarsina TR (2021). Editorial commentary: chronic anterior cruciate ligament injury requires reconstruction plus lateral tenodesis to control rotational instability: additional technical complexity may result in complications without improved outcomes. Arthroscopy.

[9] Zhang ZY, Pan XY, Maimaitijiang P (2022). Anterior tibial subluxation measured under a modified protocol is positively correlated with posterior tibial slope: a comparative study of MRI measurement methods. Knee Surg Sports Traumatol Arthrosc.

[10] Zhang ZY, Wang C, Maimaitimin M (2021). Anterior and rotational tibial subluxation in the setting of anterior cruciate ligament injuries: an MRI analysis. Knee.

[11] Zhang ZY, Huang HJ, Maimaitijiang P (2023). Comparisons of diagnostic performance and the reliability in identifying ACL injury between two measuring protocols of anterior tibial subluxation on MR images. Skeletal Radiol.

[12] Tanaka MJ, Jones KJ, Gargiulo AM (2013). Passive anterior tibial subluxation in anterior cruciate ligament-deficient knees. Am J Sports Med.

[13] McDonald LS, van der List JP, Jones KJ (2017). Passive anterior tibial subluxation in the setting of anterior cruciate ligament injuries: a comparative analysis of ligament-deficient states. Am J Sports Med.

[14] Hardy A, Klouche S, Szarzynski P, et al. A threshold value of 3.5mm of passive anterior tibial subluxation on MRI is highly specific for complete ACL tears*.* Knee Surg Sports Traumatol Arthrosc. 2019;27:885–92. 10.1007/s00167-018-5159-0 10.1007/s00167-018-5159-0 30244342

[15] Hudek R, Schmutz S, Regenfelder F (2009). Novel measurement technique of the tibial slope on conventional MRI. Clin Orthop Relat Res.

[16] Magosch A, Jacquet C, Nuhrenborger C (2022). Grade III pivot shift as an early sign of knee decompensation in chronic ACL-injured knees with bimeniscal tears. Knee Surg Sports Traumatol Arthrosc.

[17] Ahn JH, Koh IJ, McGarry MH (2021). Double-bundle anterior cruciate ligament reconstruction with lateral extra-articular tenodesis is effective in restoring knee stability in a injured knee model: a cadaveric biomechanical study. Arthroscopy.

[18] Yu PA, Fan CH, Kuo LT, et al. Differences in gait and muscle strength of patients with acute and chronic anterior cruciate ligament injury*.* Clin Biomech. 2020;80. 10.1016/j.clinbiomech.2020.105161 10.1016/j.clinbiomech.2020.105161 32961508

[19] Nguyen JT, Wasserstein D, Reinke EK (2017). Does the chronicity of anterior cruciate ligament ruptures influence patient-reported outcomes before surgery?. Am J Sports Med.

