# Supplementary material for: Chronic ACL-injured patients show increased medial and global anterior tibial subluxation measured on 1-year postoperative MR images after primary single-bundle ACL reconstruction
Source: J Orthop Surg Res. 2023 Aug 3;18:565. doi: 10.1186/s13018-023-04028-5 (PMC10401777; doi:10.1186/s13018-023-04028-5)
Supplement: Supplementary file 1 — Additional file 1. The supplementary tables and figure provide detailed data on the results of the multivariate linear regression analyses. [file 13018_2023_4028_MOESM1_ESM.pdf]

Table S1 The multivariate linear regression model for postoperative LATS.

Variables (n = 62)	Estimate (mm)	Std. Error	95% CI	*p* value
(Intercept)[a]	10.339	4.545	(1.222, 19.456)	**0.027**
Male Sex	-0.782	1.395	(-3.580, 2.016)	0.577
Age	0.076	0.085	(-0.095, 0.247)	0.375
BMI	-0.319	0.200	(-0.720, 0.082)	0.116
Beighton Score	0.191	0.254	(-0.318, 0.701)	0.454
Articular cartilage lesion	0.268	1.576	(-2.892, 3.429)	0.865
Lateral meniscal injury	1.105	1.189	(-1.280, 3.491)	0.357
Medial meniscal injury	0.347	1.529	(-2.719, 3.414)	0.821
Chronic ACL injury	1.859	1.219	(-0.587, 4.304)	0.133

ACL, anterior cruciate ligament; BMI, body mass index; LATS, lateral anterior tibial subluxation. A p value < 0.05 is indicated in bold.

[a]Intercept refers to the constant term of the linear regression model.

Table S2 The multivariate linear regression model for postoperative MATS.

Variables (n = 62)	Estimate (mm)	Std. Error	95% CI	*p* value
(Intercept)[a]	5.611	2.888	(-0.181, 11.403)	0.057
Male Sex	-3.042	0.886	(-4.819, -1.264)	**0.001**
Age	-0.035	0.054	(-0.143, 0.074)	0.526
BMI	-0.034	0.127	(-0.289, 0.221)	0.789
Beighton Score	0.158	0.161	(-0.166, 0.481)	0.333
Articular cartilage lesion	0.796	1.001	(-1.212, 2.804)	0.430
Lateral meniscal injury	0.630	0.756	(-0.885, 2.146)	0.408
Medial meniscal injury	-0.384	0.971	(-2.333, 1.564)	0.694
Chronic ACL injury	2.024	0.775	(0.470, 3.578)	**0.012**

ACL, anterior cruciate ligament; BMI, body mass index; MATS, medial anterior tibial subluxation. A p value < 0.05 is indicated in bold.

[a]Intercept refers to the constant term of the linear regression model.

Table S3 The multivariate linear regression model for postoperative GATS.

Variables (n = 62)	Estimate (mm)	Std. Error	95% CI	*p* value
(Intercept)[a]	7.975	3.434	(1.087, 14.863)	**0.024**
Male Sex	-1.912	1.054	(-4.026, 0.202)	0.075
Age	0.021	0.064	(-0.108, 0.150)	0.747
BMI	-0.177	0.151	(-0.480, 0.126)	0.247
Beighton Score	0.175	0.192	(-0.210, 0.560)	0.367
Articular cartilage lesion	0.532	1.191	(-1.856, 2.920)	0.657
Lateral meniscal injury	0.868	0.898	(-0.934, 2.670)	0.338
Medial meniscal injury	-0.019	1.155	(-2.335, 2.298)	0.987
Chronic ACL injury	1.941	0.921	(0.093, 3.789)	**0.040**

ACL, anterior cruciate ligament; BMI, body mass index; GATS, global anterior tibial subluxation. A *p* value < 0.05 is indicated in bold.

[a]Intercept refers to the constant term of the linear regression model.

Table S4 The multivariate linear regression model for postoperative IRTS.

Variables (n = 62)	Estimate (mm)	Std. Error	95% CI	*p* value
(Intercept)[a]	4.728	3.290	(-1.871, 11.327)	0.157
Male Sex	2.260	1.010	(0.234, 4.285)	**0.029**
Age	0.111	0.062	(-0.013, 0.234)	0.078
BMI	-0.285	0.145	(-0.575, 0.005)	0.054
Beighton Score	0.034	0.184	(-0.335, 0.403)	0.855
Articular cartilage lesion	-0.528	1.141	(-2.816, 1.760)	0.646
Lateral meniscal injury	0.475	0.861	(-1.251, 2.202)	0.583
Medial meniscal injury	0.732	1.107	(-1.488, 2.952)	0.511
Chronic ACL injury	-0.166	0.883	(-1.936, 1.605)	0.852

ACL, anterior cruciate ligament; BMI, body mass index; IRTS, internal rotational tibial subluxation. A *p* value < 0.05 is indicated in bold.

[a]Intercept refers to the constant term of the linear regression model.
